# Country Clergy and Secret Remedies

**Published:** 1912-01-13

**Authors:** Edgar S. Kemp


### The Editor's Letter Box.

COUNTRY CLERGY AND SECRET REMEDIES.

*To the Editor of* The Hospital.

Sir,—I think that the enclosed cutting from a Somersetshire paper may interest you in view of the paragraph on "A Royal Commission on Quackery" in The Hospital of December 30, 1911.

"The Rev C. L. Marson, Vicar of Hambridge, near Taunton, writes in 'Ecclesia' (All Saints' Parish Magazine) for January: In villages everything must be looked at in the light of history. So it is even with medicine. . . The busy practitioner has no time to educate us; and indeed, it would be a slow process. Consequently we are very much preyed upon by the vendors of secret remedies who advertise in the cheap papers. It is quite pathetic to find what large sums are spent by very poor people on pills and syrups which they have seen cried up in print. . . There is a book published by the British Medical Association (429 Strand, London), and sold for one shilling, which might well be in the hands of every country clergyman, and, indeed, in the hands of all who have dealings with the victims of the vendors of these popular medicines. It is called 'Secret Remedies; What They Cost and What They Contain.' It is not too technical for ordinary mortals to understand. It describes in simple terms what these remedies are, what they are said by the advertisers to do, what they cost and what they are worth. . . This is altogether a much more religious question than many of us seem to think."

I do not think that it is usual for the clergy to point out to their parishioners what useless and extravagant luxuries are most patent medicines. Indeed, I seem to have heard that pill proprietors and others have attempted from time to time to get the clergy to push the sale of their nostrums. I think you will agree that the Rev. C. L. Marson deserves much credit for the line he has taken.

It is interesting that next to Mr. Marson's article in the paper there is an advertisement of a well-known pill which has earned for itself a prominent position in "Secret Remedies."—Yours truly,

January 4. Edgar S. Kemp.

[Note: Owing to the great pressure on our space we have been compelled to hold over letters from the Hon. Sydney Holland, Chairman of the London Hospital, Mr. T. A. Cook, Chairman of the West Ham and Eastern General Hospital, and Dr. T. B. Rhodes, Secretary and House Governor of the North Staffordshire Infirmary.]

